# Widespread Expression of BORIS/CTCFL in Normal and Cancer Cells

**DOI:** 10.1371/journal.pone.0022399

**Published:** 2011-07-19

**Authors:** Tania A. Jones, Babatunji W. Ogunkolade, Jaroslaw Szary, Johan Aarum, Muhammad A. Mumin, Shyam Patel, Christopher A. Pieri, Denise Sheer

**Affiliations:** Queen Mary University of London, Centre for Neuroscience and Trauma, Blizard Institute, Barts and the London School of Medicine and Dentistry, London, United Kingdom; Université Paris-Diderot, France

## Abstract

BORIS (CTCFL) is the paralog of CTCF (CCCTC-binding factor; NM_006565), a ubiquitously expressed DNA-binding protein with diverse roles in gene expression and chromatin organisation. BORIS and CTCF have virtually identical zinc finger domains, yet display major differences in their respective C- and N-terminal regions. Unlike CTCF, BORIS expression has been reported only in the testis and certain malignancies, leading to its classification as a “cancer-testis” antigen. However, the expression pattern of BORIS is both a significant and unresolved question in the field of DNA binding proteins. Here, we identify BORIS in the cytoplasm and nucleus of a wide range of normal and cancer cells. We compare the localization of CTCF and BORIS in the nucleus and demonstrate enrichment of BORIS within the nucleolus, inside the nucleolin core structure and adjacent to fibrillarin in the dense fibrillar component. In contrast, CTCF is not enriched in the nucleolus. Live imaging of cells transiently transfected with GFP tagged BORIS confirmed the nucleolar accumulation of BORIS. While BORIS transcript levels are low compared to CTCF, its protein levels are readily detectable. These findings show that BORIS expression is more widespread than previously believed, and suggest a role for BORIS in nucleolar function.

## Introduction

CTCFL or BORIS (Brother of the Regulator of Imprinted Sites), a paralog of CTCF, has been classified as a cancer-testis antigen as its distribution is reported to be limited to the testis and certain cancers [1]. Abnormally high levels of BORIS transcripts are present in a variety of human tumours and cancer-derived cell lines and, in some, increased expression has been linked to promoter-specific demethylation and de-repression of co-expressed cancer-testis genes [2], [3], [4], [5], [6], [7], [8], [9], [10], [11], [12]. Different studies have reported contradictory findings of BORIS expression in some types of cancer. For example, a reported increased expression in several breast cancer cell lines and in the majority of primary breast tumours tested in one study was not confirmed by another [3], [13]. Furthermore, increased transcription levels of BORIS have been found in melanoma cell lines but not in primary melanomas [7]. Nevertheless, the importance of BORIS in cancer is suggested by the finding of high levels in advanced epithelial ovarian cancer [14].

*BORIS* and *CTCF* genes are thought to have evolved during vertebrate development from a gene duplication event [15]. Whilst the zinc finger domains that bind DNA in the respective proteins are very similar, the C- and N-terminal domains of BORIS exhibit no significant homology with CTCF or any other proteins [16]. BORIS does not contain the modular substrates for specific post-translational modifications that are critical for CTCF function and lacks the conserved C-terminal phosphorylation motif required for CTCF-mediated growth suppression. This would suggest different functions for the two proteins.

Several lines of evidence point to a role for BORIS in epigenetic regulation of gene expression. During mouse male germ-line development, BORIS is observed in primary spermatocytes but is progressively replaced by CTCF in post-meiotic germ-line cells [17]. This switch in expression from BORIS to CTCF coincides with the re-establishment of site-specific DNA-methylation patterns during male germ cell differentiation [17]. Furthermore, in tumour cell lines where CTCF silences genes by DNA methylation, conditional expression of BORIS leads to replacement of CTCF by BORIS at these genes, resulting in local demethylation and gene activation [6], [10], [11], [18].

The DNA binding and epigenetic functions attributed to BORIS would suggest a nuclear localization. However, recent reports show BORIS present mainly in the cytoplasm of prostate epithelia, testis and prostate cancer cell lines [1] while nuclear localization is only identified at specific stages of spermatogenesis and in some 5-aza-dexoxycytidine-treated prostate cancer cell lines [5]. Here, we show predominant localization of BORIS within the nucleolus in several cancer cell lines and primary cells, with enrichment within the nucleolin core structure and adjacent to fibrillarin in the dense fibrillar component.

## Results and Discussion

### BORIS expression in normal and cancer cells and tissues

**Figure 1:**
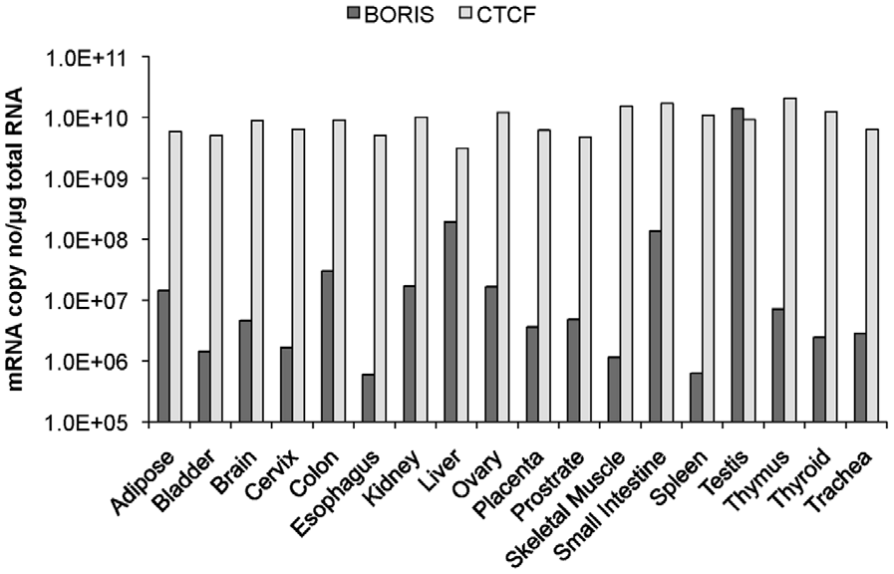
BORIS and CTCF expression in normal human tissues. BORIS transcript levels in normal human tissues (First choice® Human Total RNA Survey Panel, Ambion, UK). Error bars represent the standard deviation.

To determine the level of BORIS expression in normal human tissues, total RNA was obtained from human adipose, bladder, brain, cervix, colon, esophagus, kidney, liver, ovary, placenta, prostate, skeletal muscle, small intestine, spleen, testis, thymus, thyroid and trachea (Ambion). Real-time RT-PCR showed the highest BORIS transcript levels in the testis (10^10^ transcripts/µg total RNA), while lower levels were detected in the other tissues (10^5^-10^8^ transcripts/µg total RNA) in agreement with other reports [7] (Fig. 1). We did not detect BORIS in heart or lung, although this may be due to a limitation in the sensitivity of our assay. CTCF transcript levels were relatively constant in all tissues (10^9^–10^10^ transcripts/µg total RNA) (Fig 1).

**Figure 2:**
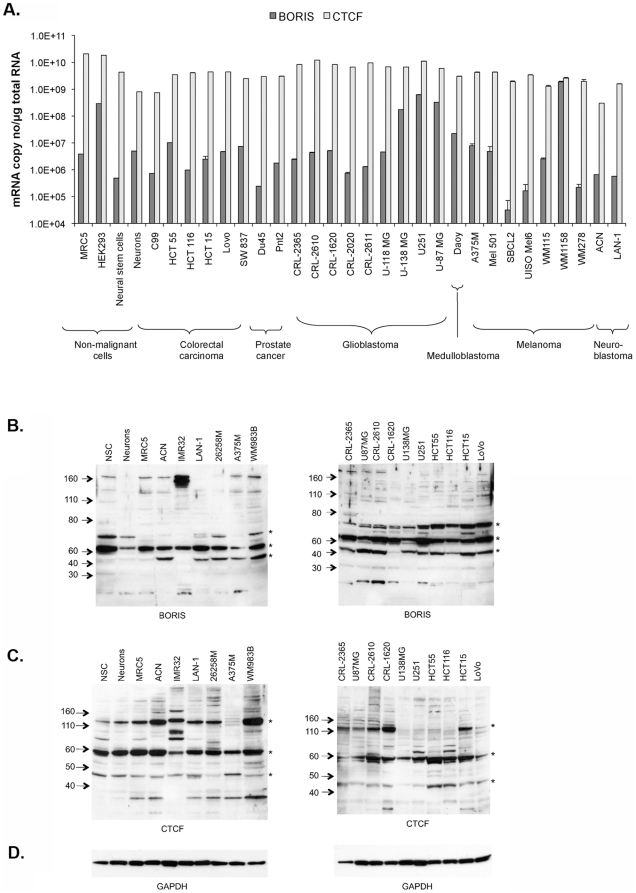
BORIS and CTCF expression in normal and cancer cell lines. **A**, BORIS transcript levels in selected cell lines. **B**, Western blotting for BORIS showing major bands (*) at approximately 76 KDa (BORIS theoretical size), 60 KDa and 45 KDa. The smaller bands may represent BORIS isoproteins as described recently [19]. **C**, Western blotting for CTCF showing major bands (*) at approximately 130 kDa, 60 KDa and 48 KDa. These different bands may represent differentially expressed CTCF isoforms as described recently [36]. BORIS and CTCF are present in normal cells (neurons, neural stem cells (NSC) and MRC5 fetal lung fibroblasts), neuroblastoma cell lines (ACN, IMR32 and LAN-1), melanoma cell lines (26258M, A375M and WM983B), glioblastoma cell lines (CRL-2365, U87MG, CRL-2610, CRL-1620, U138MG and U251) and colorectal cell lines (HCT55, HCT116, HCT15 and LoVo). **D**, GAPDH was used as a control for loading differences. Invitrogen Novex®Sharp Pre-Stained Protein Standard, LC5800, was used for determination of band size. Error bars in (A) represent the standard deviation.

Next, we compared transcript levels of BORIS and CTCF in a variety of normal and cancer cell lines. Real-time RT-PCR analysis showed similar levels of BORIS and CTCF mRNA in fibroblasts, embryonic kidney cells, neural stem cells, neurons, colorectal, prostate, medulloblastoma, glioblastoma, melanoma and neuroblastoma cell lines to those observed in human tissues. BORIS expression was lower (10^6^–10^7^ transcripts/µg total RNA), compared to the more abundant CTCF (10^9^–10^10^ transcripts/µg total RNA) (Fig. 2A). Hence, BORIS and CTCF are present in both normal and cancer cells, with expression of CTCF often 1000 fold greater.

Unexpectedly, Western blotting showed similar levels of BORIS expression in normal and tumour cell lines (Fig. 2B). We identified strong major bands of 60–70 kDa in agreement with the theoretical molecular weight of BORIS [19]. We also detected some lower and higher molecular weight bands. It is not yet clear which of these bands corresponds to the recently described isoforms of BORIS [19] or which are BORIS with post-translational modifications. However, incubating the BORIS antibody with BORIS peptide completely blocked all bands (Fig. S1). None of these BORIS reactive bands were detected when the membranes were probed with CTCF antibodies (Fig. 2C).

To confirm the specificity of the BORIS antibody, HEK293T cells were transfected with GFP-tagged BORIS, or GFP-tagged CTCF constructs. Western analysis confirmed the presence of BORIS protein in cells transfected with GFP-tagged BORIS, whilst only endogenous BORIS was detected in untransfected cells or cells transfected with GFP-CTCF or empty vector (Fig. S2).

**Figure 3:**
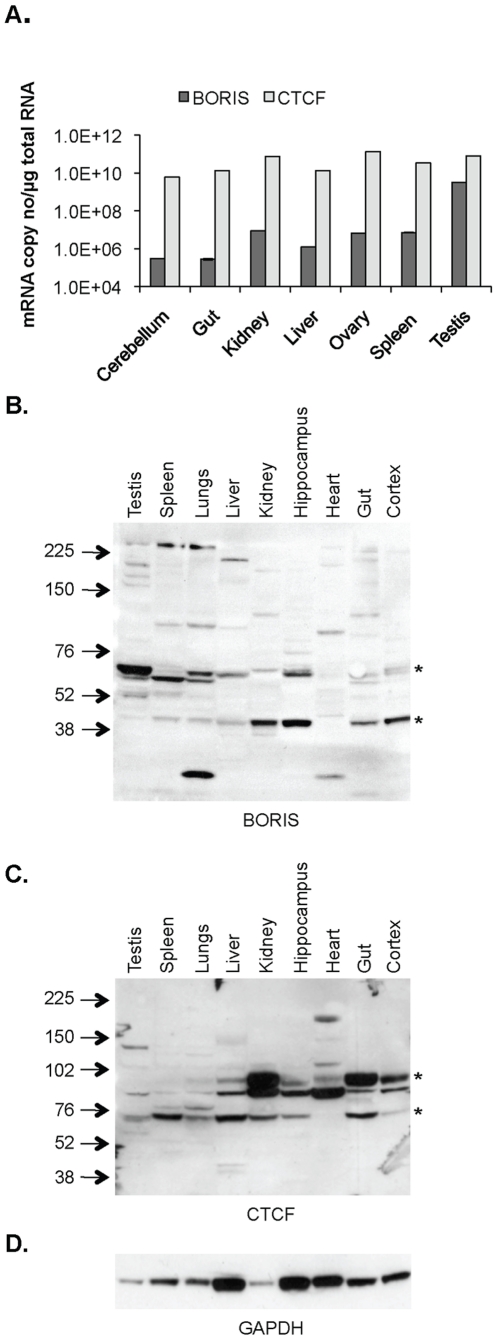
BORIS and CTCF expression in mouse tissues. **A**, BORIS and CTCF mRNA levels in selected normal mouse tissues. **B**, Western blotting for BORIS showing major bands (*) at 60–70 KDa and 45 KDa in mouse tissues. **C**, Western blotting for CTCF showing bands (*) between 70–100 KDa in mouse tissues. **D**, GAPDH was used as a control for loading differences. GE Healthcare Full Range Rainbow Marker, RPN800E, was used for determination of band size. Error bars in (A) represent the standard deviation

We then used real-time RT–PCR to determine the levels of BORIS expression in normal mouse tissues including cerebellum, gut, kidney, liver, ovary, spleen and testis. As in human tissues, we found a far lower level of BORIS compared to CTCF in mouse tissues with the exception of the testis (Fig. 3A). Western blotting on a selection of mouse tissues revealed several bands, the most abundant of which were at 60–70 kDa (Fig. 3B). We also detected a band of approximately 48 kDa in the kidney, hippocampus, gut and the cortex, whilst other less intense bands above 200 kDa were present in the testis, spleen, lungs and the liver (Fig. 3B). These higher molecular weight bands may correspond to homo/heterodimer of BORIS, or poly(ADP)-ribosylation as has been described for CTCF [20]. Again, no bands were detected when membranes were probed with BORIS antibodies pre-incubated with free BORIS peptides (Fig. S3), neither did we detect the same bands when membranes were probed with CTCF antibodies (Fig. 3C). Although BORIS protein is clearly detectable, we only detect low levels of BORIS transcripts, both in cells and tissues (Fig 1, Fig 2A and Fig 3A). However, discordance between mRNA level and protein abundance has been reported to be poor for certain other genes [21], [22], [23].

In view of the similarity in their zinc finger domains, BORIS has been suggested to be an antagonist, competitor or regulator of CTCF in germ cells and human cancers [17]. Indeed, BORIS and CTCF do compete for binding to common DNA targets. For example, CTCF occupancy at the *MAGE-A1* promoter only occurs if BORIS expression levels are low [11]. As the N- and C-terminal ends of BORIS and CTCF are different, they are likely to be responsible for different functional outcomes, such as mediating DNA demethylation, after binding to DNA. The relative levels of BORIS and CTCF are thus likely to be crucial for maintaining stability in normal gene expression profiles.

### Nucleolar localization of BORIS

**Figure 4:**
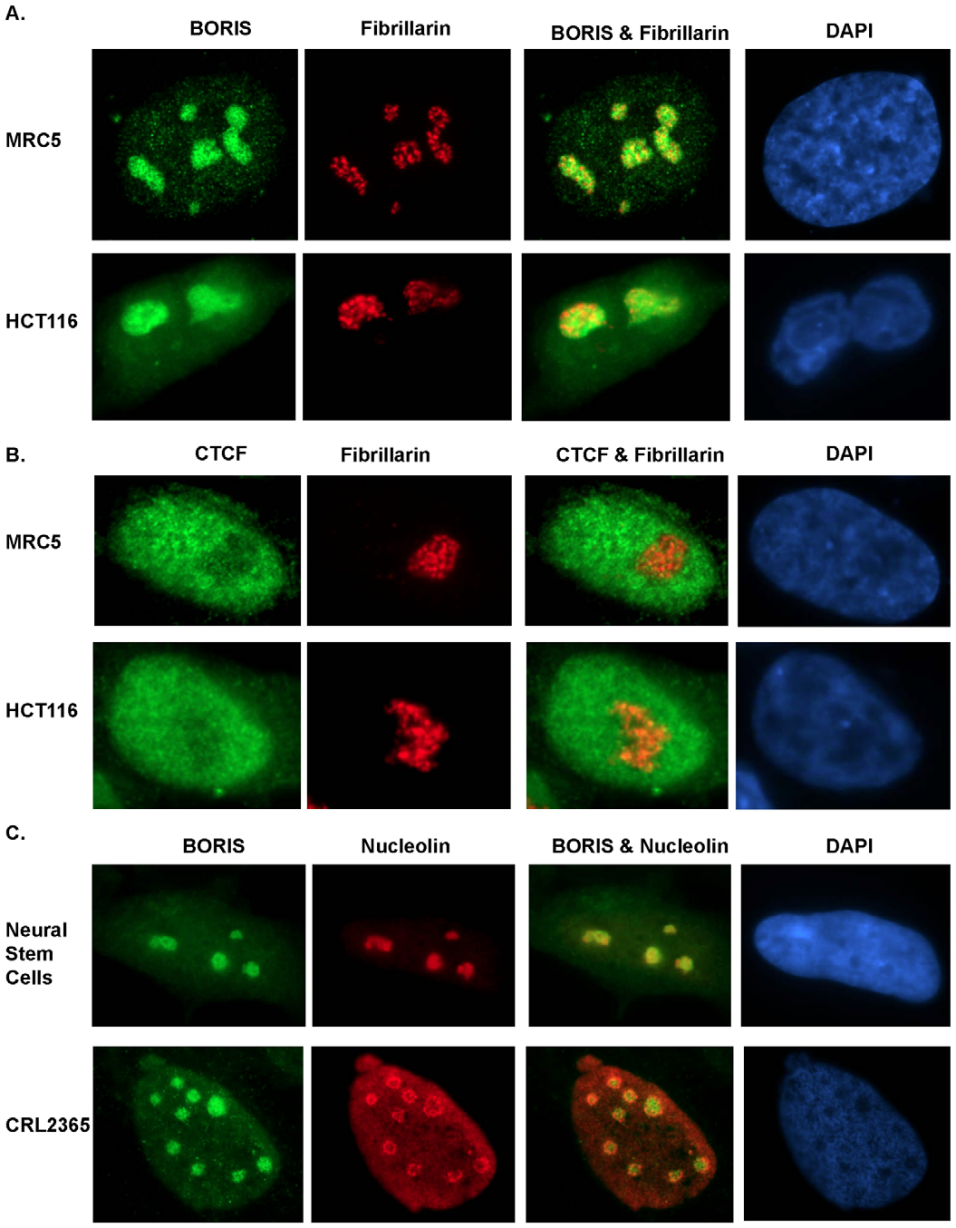
Cellular distribution of BORIS in human cell lines and tissues. **A, B** Immunofluorescence imaging of BORIS or CTCF staining in green (Alexa 488) together with fibrillarin staining in red (Alexa 594) in MRC5 and colorectal cell line HCT116 showing enrichment of BORIS, but not CTCF, within the nucleolus. Cells were counterstained with 4′, 6-Diamidino-2-phenylindole (DAPI) shown in blue. BORIS and fibrillarin are close neighbours with each other and the dense fibrillar component (DFC) of the nucleolus. Images are shown at 100x magnification. **C** Double immunostaining showing BORIS staining in green (Alexa 488) within the Nucleolin region (red, Alexa 594) in human neural stem cells and glioblastoma cell line CRL2365. Cells were counterstained with 4′, 6-Diamidino-2-phenylindole (DAPI) shown in blue. Images are shown at 100x magnification.

**Figure 5:**
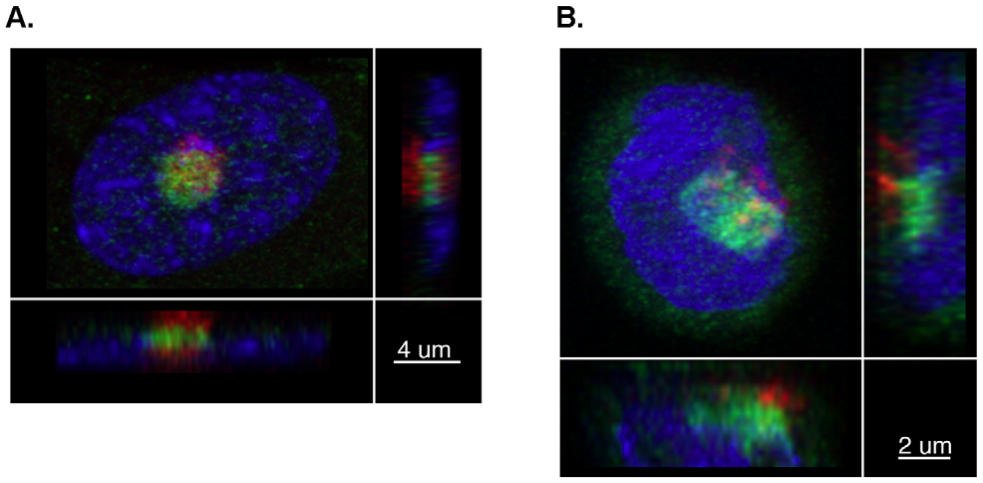
BORIS is adjacent to fibrillarin in the nucleolus. Three-dimensional confocal images showing BORIS staining in green (Alexa 488) and fibrillarin staining in red (Alexa 594). Cells are counterstained with 4′, 6-Diamidino-2-phenylindole (DAPI, blue). **A**, MRC5 fibroblasts and **B**, C99 colorectal cell line. Images are shown at 63x magnification.

Immunofluorescence staining showed that BORIS is located within the cytoplasm and the nucleus of several human cell types, including MRC5 fibroblast, HEK293T embyonic kidney cells, neural stem cells, colorectal, neuroblastoma, melanoma, prostate and glioblastoma cell lines. In all cell types examined, BORIS immunoreactivity was enriched in the nucleolus (Fig. 4 and Fig. S4). Overnight incubation of BORIS antibody with the peptide completely blocked the immunofluorescent signal further confirming the specificity of the BORIS antibodies (Fig. S5). No immunoreactivity was detected when cells were stained with non-specific IgG antibodies. Co-staining of cells for BORIS or CTCF and fibrillarin, a highly conserved protein that is associated with small nucleolar RNAs [24], confirmed enrichment of BORIS but not CTCF in the nucleolus (Fig. 4A,B). Further co-staining showed BORIS within the nucleolar space occupied by nucleolin, an abundant non-ribosomal nucleolar protein [25] (Fig. 4C). Confocal microscopy and 3D reconstruction confirmed that BORIS was within the nucleolus, adjacent to fibrillarin in both normal and cancer cells (Fig. 5A, B and Movies S1, S2, S3 and S4).

**Figure 6:**
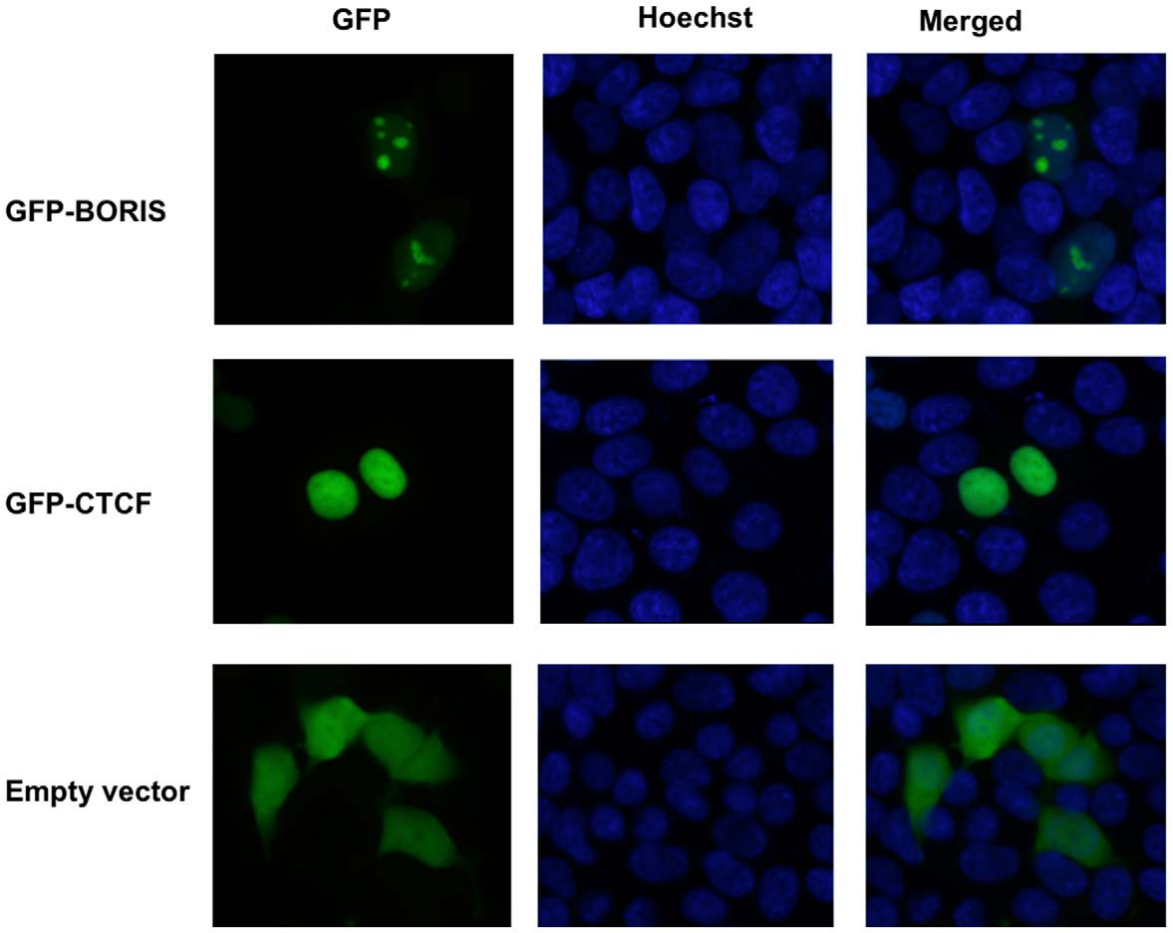
Transfection of BORIS confirms enrichment in the nucleolus. Live cell imaging of HEK293T cells transiently transfected with GFP-tagged BORIS (green), GFP-tagged CTCF (green) or empty vector (green), counterstained with Hoechst 333412 (blue). Images are shown at 40x magnification.

To confirm these findings, HEK293T cells were transfected with GFP-tagged BORIS or GFP-tagged CTCF constructs. Using live-cell imaging, GFP was found to accumulate progressively in nucleoli of cells transfected with GFP-BORIS. 71.3% +/− 1.7 of GFP-BORIS transfected cells showed clear nucleolar localization (873 cells analysed in total from two independent experiments). In contrast, cells transfected with GFP-CTCF showed an even distribution of GFP throughout the nucleus, and those cells transfected with an empty GFP vector showed mainly GFP in the cytoplasm (Fig. 6). Furthermore, immunostaining of cells 72 hours after transfection of HEK293T cells with BORIS specific shRNA plasmids, but not empty vector, significantly (*p*<0.05) reduced the nucleolar immunostaining of BORIS (Fig. S6A, B). This was accompanied by a corresponding reduction in BORIS transcript levels (Fig S7).

### Putative Nucleolar Localization Sequences

Computational analysis of the BORIS protein sequence Q8NI51, [26], revealed 2 putative nucleolar localization sequences in the zinc finger domain:

- LIQHQKTHKNEKRFKCKHCSYACKQ (between positions 473 and 497) (ZF6/7)
- AKSAASGKGRRTRKRKQTILKEATKGQK (between positions 573 and 600) (ZF9/10)

Five putative nucleolar localization sequences were predicted in CTCF, three of which are in the N terminal and overlap with the CTCF K/R acetylation sites predicted by Klenova, et al. [17]. The predicted nucleolar localization sequences in CTCF are as follows:

- ESETFIKGKERKTYQRRREGGQEE (between positions 12 and 35)
- KDPDYQPPAKKTKKTKKSKLRYTEEGKDV (between positions 193 and 221)
- VGNMKPPKPTKIKKKGVKKTFQCEL (between positions 246 and 270) (N terminal)
- GENGGETKKSKRGRKRKMRSKKEDSSDSENA (between positions 585 and 615) (ZF 9/10)
- QPVTPAPPPAKKRRGRPPGRTNQPKQNQPTAI (between positions 639 and 670).

These sequences would support the finding of BORIS in the nucleolus as well as the translocation of CTCF into the nucleus during differentiation of human hematopoietic cells [27].

UBF (upstream binding factor) has been recently identified as the first common interaction partner of BORIS and CTCF [28]. This interaction was shown to be direct and to require the zinc finger domains of BORIS and CTCF, the high mobility group (HMG)-box 1, and the dimerization domain of UBF. CTCF was found to bind immediately upstream of the ribosome spacer promoter in a methylation-sensitive manner, where it is suggested to load UBF onto rDNA to form part of a network that maintains rDNA genes poised for transcription. Our immunofluorescence staining did not show enrichment of CTCF in the nucleolus, however, low levels of CTCF may be present but below detection in our assay. Torrano et al., demonstrated that the translocation of CTCF to the nucleolus after induction of differentiation in human and rat cells was regulated by poly(ADP-ribosyl)ation [27]. UBF is very abundant in the nucleolus and has been shown to be highly dynamic [29]. Thus, the direct interaction between CTCF and UBF may be transient and, since BORIS recognizes the same DNA binding sites [1], there may be competition between CTCF and BORIS to bind UBF [22]. Taken together with the findings described here, the direct interaction of BORIS with UBF suggests BORIS plays an important role in ribosome biogenesis.

### Concluding remarks

This is the first report showing BORIS localising predominantly in the nucleolus in many different malignant and non-malignant cell types. The nucleolus is a multi-functional sub-nuclear compartment where for example, ribosomal RNAs are synthesized, processed and assembled with ribosomal proteins [30], [31], [32]. However, extensive proteomic analysis has revealed the dynamic nature of the nucleolar proteome and suggests that the nucleolus may perform many other biological roles in addition to ribosome biogenesis such as the regulation of specific aspects of mitosis and cell cycle progression [24], [33], [34]. Here we demonstrate that BORIS is clearly more than a testis-specific protein, and show a distinct subcellular localization in multiple cell lines as well as normal human and mouse tissues. BORIS is enriched in the nucleolus, inside nucleolin core structure and adjacent to fibrillarin. Our novel finding of widespread expression of BORIS together with its nucleolar localization warrants further investigation to ascertain its precise function in this context.

## Materials and Methods

### Cell Culture

Cancer cell lines were maintained in RPMI or DMEM supplemented with 10% fetal bovine serum (unless stated otherwise), 100-units/ml penicillin, 100-mg/ml streptomycin, and 0.29 mg/ml L-glutamine (Invitrogen). Cell lines used were human lung fibroblasts MRC5 (ECACC); embryonic kidney cell line HEK293T; colorectal cell lines C99, HCT55, HCT116, HCT15, LoVo and SW837; medulloblastoma cell line DAOY; prostate cancer cell lines Du45 and Pnt2; melanoma cell lines A375M, Mel 501, SBCL2, UISO, Mel 6, WM115, WM1158, M2629bM and WM278; neuroblastoma cell lines ACN, IMR-32 and LAN1 and glioblastoma cell lines CRL-2365, CRL-2610 (LN-18), CRL-1620 (A-172), CRL-2020, CRL-2611, U-118MG, U-138 MG, U251 and U87 MG (ATCC). MRC5 cells were grown in the above media, supplemented with 20% fetal bovine serum. Human neural stem cells derived from the cell line H9 (46,XX) (EnStem-A, Millipore) were cultivated in Neurobasal medium (Invitrogen, 21103-049) supplemented with B27 (Invitrogen, 12587-010), FGF-2 10 ng/ml (PeproTech), 1% penicillin/streptomycin (Invitrogen) and 2 mM glutamine (Invitrogen). Half the medium was changed every other day. In vitro differentiation was induced by omitting FGF-2 from the medium.

### Tissue protein isolation

Mouse tissues were isolated from 3-month-old C57bl6 or NOD mice and snap frozen in liquid nitrogen. All experimental protocols involving mouse tissues were approved by the Home Office Animal Procedures Committee, under the project license number PPL 70/6693. Tissues were disrupted in Qiagen RLT lysis buffer for RNA extraction, or into 1X RIPA buffer (50 mM Tris-HCl pH 8.0, 150 mM NaCl, 1% NP-40, 0.5% Na Deoxycholate, 0.1% SDS) containing protease inhibitors (Protease Inhibitor Cocktail Set III EDTA-free, Calbiochem) and phosphatase inhibitors (Phosphatase Inhibitor Cocktail Set II, Calbiochem) for protein analysis. Total protein lysates were prepared from 10^8^ cells in 1X RIPA buffer. Lysed cells were sonicated briefly with a Bioruptor and debris cleared by centrifugation. The protein content of the extracts was estimated using BCA kit (Thermo Fisher Scientific) according to the manufacturer's protocol.

### RNA Isolation and Reverse Transcription

Total RNA was isolated using silica-based spin-column extraction kit (RNeasy mini kit, Qiagen) following the manufacturer's protocol. Total RNA was treated with RNase-free DNase1 (Ambion) to reduce genomic DNA contamination. RNA integrity was evaluated using the Agilent Bioanalyzer. Two micrograms of total RNA was reverse transcribed with SuperScriptase III (Invitrogen) using Oligo-dT primers or random hexamers according to the manufacturer's protocol. Negative (-RT) controls contained RNase-free water substituted for reverse transcriptase.

### Quantitative Real-Time PCR

Both the published primers [3] and our own designed with Primer Express 2.0 were used in this study. Together these primers amplify 19 out of the 23 isoforms of BORIS described recently [19]. Human primers were as follows:

BORIS exon 4-5 forward:

5′-AAAACCTTCCGTACGGTCACTCT-3′

and reverse: 5′-TGTTGCAGTCGTTACACTTGTAGG-3′

and probe: Fam-TAACACCCACACAGGAACCA-Tam

CTCF exon 5-6 forward: 5′-GAGAAGCCATTCAAGTGTTCCAT-3′

and reverse: 5′-CTCCAGTATGAGAGCGAATGTGA-3′

and probe: Fam-ATTACGCCAGTGTAGAAGTCAGC-Tam.

Mouse primers were as follows:

BORIS exon 5–6 forward:

5′-AGTGCTCCCTGTGCAAGTACG-3′

and reverse 5′-GTAAGCACACTGGCAACACTGG-3′

and probe: Fam-AAGCAAGATGAAGCGTCACAT-Tam

CTCF exon 5–6 forward:

5′-TCGTTATAAACACACTCATGAGAAACC-3′

and reverse: 5′-TCTCCAGTATGAGAGCGAATGTG-3′

and probe: Fam-AGTGTTCCATGTGTGATTGTCAG-Tam.

mRNA levels were quantified on an ABI7500 instrument using SYBR Green JumpStart Taq ReadyMix kit (Sigma-Aldrich) or platinium Taq polymerase kit (Invitrogen) with 50–100 ng of cDNA (except for BORIS primers when 150–200 ng of cDNA was used) and 100–200 nM primers. We used primers spanning the exon 4/5 junction of BORIS and findings were confirmed using published primers to exon 6/7 [2], [3], and exon 9/10 [13] in a qRT-PCR assay with various concentrations of total cellular RNA. Absolute concentrations were estimated using standard curves generated from serial dilution of amplicons. The threshold cycle from serial dilutions of single stranded oligonucleotides was plotted against the log copy numbers of the target PCR products, and reported as copy numbers/µg of total RNA [35].

### Western Blot Analysis

20–50 µg of protein extract and 10 ul of molecular weight standard (Invitrogen Novex®Sharp Pre-Stained Protein Standard, LC5800 or GE Healthcare Full Range Rainbow Marker, RPN800E) were separated on a 4–12% gradient NuPAGE polyacrylamide gel (Invitrogen) and then blotted onto nitrocelluose membrane (Invitrogen) according to the manufacturer's protocol. The nitrocellulose membrane was incubated in Tris-buffered saline blocking solution containing 5% skimmed milk and 0.1% Tween-20. The membrane was then probed with primary antibodies using standard conditions. We selected two antibodies against BORIS; a rabbit polyclonal antibody ab18377 (Abcam), raised against a synthetic peptide within the first 100 amino acids, and a rabbit polyclonal antibody HPA001472 (Sigma), raised against amino acids 33–172. These commercially available antibodies should recognize most of the 23 isoforms of BORIS and have been characterized previously [13], [28]. BORIS antibody ab18337 (1∶1000 dilution, Abcam) was used for all Western data shown and BORIS antibody HPA001472 (1∶200 dilution, Sigma) was used to confirm the bands (data not shown). For CTCF, we used antibody 07–729 (1∶1000 dilution, Millipore) and for GAPDH we used antibody 14C10 (1∶2000 dilution, Cell Signalling). After several washes, bands were revealed with the corresponding horseradish peroxidase coupled secondary antibody and detected using the ECL detection kit (GE Healthcare) according to the manufacturer's protocol.

### Immunofluorescence

Cells were grown on coverslips and fixed with 4% paraformaldehyde (Electron Microscopy Sciences) in phosphate-buffered saline, 0.14 M NaCl, 2.7 mM KCl, 8.1 mM Na2HPO4, 1.5 mM KH2PO4, pH 7.2–7.4 for 10 minutes at room temperature, followed by permeabilization in blocking buffer (phosphate-buffered saline containing 0.1% Triton X-100, 0.01% saponin and 10% goat serum) for 30 minutes at room temperature. Cells were then incubated with the specific antibodies in blocking buffer overnight at 4°C. BORIS antibody ab18337 (1∶100 dilution, Abcam) was used for all standard imaging. BORIS antibody HPA001472 (1∶25 dilution, Sigma) was used for confocal imaging and movies. Staining with both antibodies showed identical localization. We used CTCF antibody 07–729 (1∶1000 dilution, Millipore), Nucleolin (C23) antibody sc-8031 (1∶1000 dilution, Santa Cruz Biotechnology) and Fibrillarin antibody ab18380 (1∶1000 dilution, Abcam). Cells were washed in PBS, and primary immunoreactions visualized after incubation for 1 hour at room temperature with the Alexa Fluor 594 chicken anti-mouse IgG, A-21201 and Alexa Fluor 488 chicken anti-rabbit IgG, A-21441 (both Invitrogen). Nuclei were counterstained with 0.1 mg/ml 4′, 6-Diamidino-2-phenylindole (DAPI, Molecular Probes) and coverslips were mounted in Mowiol (Calbiochem). For standard 2 dimensional analysis specimens were visualized using a Zeiss Axiophot microscope equipped for epifluorescence using Zeiss plan-neofluar 20x, 40x or 100x objectives. Separate grey-scale images were recorded with a cooled CCD-camera (Hamamatsu, Welwyn Garden City, UK). Image analysis was performed using SmartCapture X software (Digital Scientific, Cambridge, UK). For 3 dimensional analysis specimens were studied using a Zeiss Meta 510 LSM using a 63x objective. Deconvolution of images was performed using Huygens Essential software and images then processed in Imaris x64 (v7.1.1).

### Peptide Competition

Antibody specificity was assessed using a specific blocking peptide ab22203 (Abcam). 20 ug peptide was added to 10 ug BORIS antibody ab18337 (Abcam) in 200 ul TBST buffer and incubated at 37°C overnight. Both blocked and unblocked antibody was diluted 1∶10 in blocking buffer for immunofluorescence. All images for both blocked and unblocked antibody were collected using the same exposure times. BORIS antibody specificity was also assessed by performing Western blotting with peptide blocked antibody diluted 1∶100 in blocking solution containing 2 ug/ml blocking peptide. Control unblocked antibody was incubated at the same time in appropriate blocking buffer without the addition of blocking peptide.

### Cloning and Transfection

Recombinant constructs GFP-BORIS or GFP-CTCF, were prepared by inserting BORIS or CTCF cDNA into pEGFP-C3 vector (Clontech). Fragments encoding full length BORIS (1–663aa) and CTCF (1-727) were generated by PCR using the following primers:

BORIS-GFP forward:

5′-CGGAATTCTGATGGCAGCCACTGAGATCTCTGTC-3′

and reverse: 5′-GCGGGATCCTCACTTATCCATCGTGTTGAGGAGC-3′

CTCF-GFP forward:

5′-CGGAATTCTGATGGAAGGTGATGCAGTCGAAGCC-3′

and reverse: 5′-GCGGGATCCTCACCGGTCCATCATGCTGAGAGGATC-3′

and pCMV6-XL5-BORIS or pCMV6-XL5-CTCF plasmids (Origene) as templates, respectively.

PCR fragments were digested with EcoRI/BamHI inserted into the pEGFP-C3 MCS in-frame of EGFP. Each construct was confirmed by sequencing. The resulting GFP-BORIS, GFP-CTCF and pEGFP-C3 vectors were transfected into HEK293T cells using FuGene 6-HD (Roche) according to manufacturer's protocol. Live transfected cells plated onto glass coverslips were stained with Hoechst 333412 (Invitrogen) and imaged 48 hours after transfection. For Western analysis, three million cells were transfected using FuGene 6-HD (Roche) and cells collected for protein extraction 72 hours later.

### shRNA mediated knockdown of BORIS

HEK293T cells were transiently transfected using purified and sequence verified plasmids: GI320730 (targeted to a BORIS specific site encoded by Exon 5 which is included in 19/23 splice variants identified by Pugacheva et al., [19]), GI320731 (targeted to a BORIS specific site encoded by Exon 3 which is present in all 23 variants), TR30007 (empty pGFP-V-RS vector) and TR30008 (29-mer Non-Effective GFP (pGFP-V-RS)) from Origene (kit catalogue number TG305184). Briefly, HEK293T cells were transfected with shRNA clones GI320730, GI320731, TR30008 or TR30007 using FuGene 6-HD (Roche). Cells were analysed 72 hours after transfection. Three biological replicate shRNA experiments were performed for each shRNA clone. Quantification of mRNA transcripts was performed using RT-PCR and all data normalized to GAPDH and empty vector set to 1. For quantification of BORIS immunofluorescence staining, images of HEK293T cells were collected 72 hours after transfection using the same exposure time. Images captured from 5 random fields containing at least 30 cells were imported into ImageJ software version 1.44 (Rasband, W.S., ImageJ, U.S. National Institutes of Health, Bethesda, Maryland, USA, http://imagej.nih.gov/ij/). Regions of interest (ROI) were created as a mask around DAPI stained nuclei (blue) and the threshold adjusted to remove background. Fluorescence intensity was calculated for BORIS Alexa 594 signal (red) within this mask and mean nuclear fluorescence was calculated by dividing the total intensity by nuclear area in each image. Data were exported into Excel and statistical significance was assessed by One-Way Anova analysis.

## Supporting Information

Figure S1 **Antibody peptide competition in normal and cancer cell lines.** Western blot analysis using BORIS antibody ab18337 (Abcam) with and without specific blocking peptide ab22203 (Abcam). Incubating 20 ug peptide with 10 ug BORIS antibody ab18337 (Abcam) in 200 ul TBST buffer at 37°C overnight completely blocked the bands obtained using un-blocked antibody. Both blocked and un-blocked BORIS antibody was used at a dilution of 1∶100 in blocking buffer containing 2 ug/ml of peptide.
(TIF)

Figure S2 **Western confirmation of BORIS antibody specificity.** HEK293 cells transiently transfected with GFP-BORIS, GFP-CTCF or pEGFP-C3 empty vector (Clontech). Membranes probed with GFP, BORIS, CTCF or GAPDH antibodies. GFP-BORIS migrates at approximately 100 KDa, GFP-CTCF migrates at 140 KDa and GFP alone at 30 KDa. In comparison to the strong bands detected for GFP-BORIS, endogenous BORIS migrates at 50–70 KDa as much weaker bands. GAPDH was used as a control for loading differences. Invitrogen Novex®Sharp Pre-Stained Protein Standard, LC5800, was used for determination of band size.
(TIF)

Figure S3 **Antibody peptide competition in mouse tissues.** Western blot analysis using BORIS antibody ab18337 (Abcam) with and without specific blocking peptide ab22203 (Abcam). Incubating 20 ug peptide with 10 ug BORIS antibody ab18337 (Abcam) in 200 ul TBST buffer at 37°C overnight completely blocked the bands obtained using un-blocked antibody. Both blocked and un-blocked BORIS antibody was used at a dilution of 1∶100 in blocking buffer containing 2 ug/ml of peptide.
(TIF)

Figure S4 **Distribution of BORIS in various cell lines.** Immunofluorescence imaging of BORIS in green (Alexa 488) together with fibrillarin in red (Alexa 594), counterstained with 4′, 6-Diamidino-2-phenylindole (DAPI) in blue showing enrichment of BORIS within the nucleoli of **A**, neuroblastoma cell lines, **B**, colorectal cell lines, **C**, prostate cancer cell line and **D**, melanoma cell lines. Images are shown at 100x magnification.
(TIF)

Figure S5 **Antibody peptide competition in MRC5 cells.** Immunofluorescence imaging of BORIS in red (Alexa 594) without peptide competition (**A**) and peptide neutralised BORIS antibody (**B**) counterstained with 4′, 6-Diamidino-2-phenylindole (DAPI) in blue. Images were obtained using identical exposure times and processing for a direct comparison of antibody versus antibody-peptide competition. Both blocked and un-blocked BORIS antibody was used at a dilution of 1∶10 in blocking buffer containing 2 ug/ml of peptide. Images are shown at 100x magnification.
(TIF)

Figure S6 **Immunofluorescence staining of HEK293T cells after BORIS knockdown. A**, HEK293T cells transiently transfected with BORIS-specific shRNA, GI320730 (Exon 5 in NM_080618.2) or GI320731 (Exon 3, NM_080618.2), empty vector (TR30007) or scrambled control (TR30008). shRNA vectors express GFP (green) as a transfection reporter and cells were stained for BORIS (red) 72 hours after transfection. Cells were counterstained with 4′, 6-Diamidino-2-phenylindole (DAPI) shown in blue. Note the almost complete absence of nucleolar BORIS in GI320730 and GI320731 transfected cells (green). Images are shown at 40x magnification. **B**, Quantification of nuclear BORIS in A. Data is expressed as mean nuclear intensity (arbitrary units, AU) of all cells in 5 random fields with at least 30 cells per field and represents the data from 3 biological repeats. p-values in B were calculated by one-way ANOVA as compared to nuclear intensity in cells transfected with scrambled control. Error bars represent the standard deviation.
(TIF)

Figure S7 **Transient knockdown of BORIS in HEK293T cells.** Quantification of BORIS and CTCF transcripts in HEK293T cells after transient transfection with with BORIS-specific shRNA, GI320730 (Exon 5 in NM_080618.2) or GI320731 (Exon 3, NM_080618.2) or empty vector (TR30007). Data is normalised to GAPDH and empty vector set to 1. Error bars represent the standard deviation.
(TIF)

Movie S1 **MRC5 fibroblast nucleus stained with BORIS in green (Alexa 488) and fibrillarin in red (Alexa 594), with 4′, 6-Diamidino-2-phenylindole (DAPI) counterstaining in blue.** BORIS immunoreactivity is enriched in the nucleolus adjacent to fibrillarin. Optical sections were obtained by confocal microscopy using a Zeiss Meta 510 LSM, and three-dimensional reconstruction performed using Imaris x64 (v7.1.1). Images are shown at 63x magnification.
(AVI)

Movie S2 **MRC5 fibroblast nucleus stained with BORIS in green (Alexa 488) and fibrillarin in red (Alexa 594), with 4′, 6-Diamidino-2-phenylindole (DAPI) counterstaining in blue.** BORIS immunoreactivity is enriched in the nucleolus adjacent to fibrillarin. Optical sections were obtained by confocal microscopy using a Zeiss Meta 510 LSM, and three-dimensional reconstruction performed using Imaris x64 (v7.1.1). Surface reconstruction of each channel was performed with colour properties and transparency adjusted to show spatial distribution of BORIS with respect to fibrillarin in the nucleolus. Images are shown at 63x magnification.
(AVI)

Movie S3 **Nucleus from a colorectal cell line, C99, stained with BORIS in green (Alexa 488) and fibrillarin in red (Alexa 594), with 4′, 6-Diamidino-2-phenylindole (DAPI) counterstaining in blue.** BORIS immunoreactivity is enriched in the nucleolus adjacent to fibrillarin. Optical sections were obtained by confocal microscopy using a Zeiss Meta 510 LSM and three-dimensional reconstruction performed using Imaris x64 (v7.1.1). Images are shown at 63x magnification.
(AVI)

Movie S4 **Nucleus from a colorectal cell line, C99, stained with BORIS in green (Alexa 488) and fibrillarin in red (Alexa 594), with 4′, 6-Diamidino-2-phenylindole (DAPI) counterstaining in blue.** BORIS immunoreactivity is enriched in the nucleolus adjacent to fibrillarin. Optical sections were obtained by confocal microscopy using a Zeiss Meta 510 LSM and three-dimensional reconstruction performed using Imaris x64 (v7.1.1). Surface reconstruction of each channel was performed with colour properties and transparency adjusted to show spatial distribution of BORIS with respect to fibrillarin in the nucleolus. Images are shown at 63x magnification.
(AVI)
