# Supplementary figures and images for: Widespread Expression of BORIS/CTCFL in Normal and Cancer Cells
Source: PLoS One. 2011 Jul 19;6(7):e22399. doi: 10.1371/journal.pone.0022399 (PMC3139640; doi:10.1371/journal.pone.0022399)

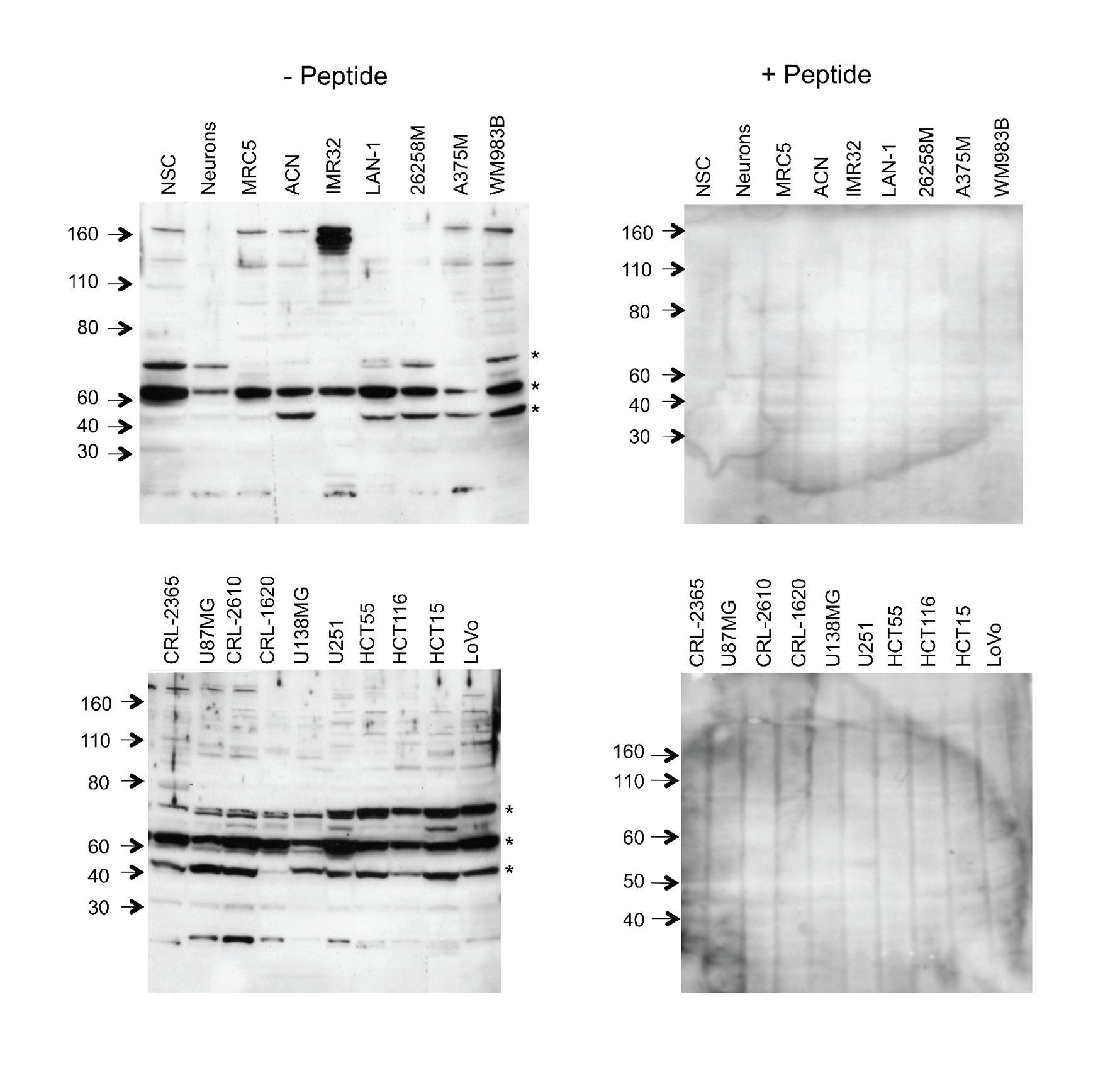

Supplement: Figure S1 — Antibody peptide competition in normal and cancer cell lines. Western blot analysis using BORIS antibody ab18337 (Abcam) with and without specific blocking peptide ab22203 (Abcam). Incubating 20 ug peptide with 10 ug BORIS antibody ab18337 (Abcam) in 200 ul TBST buffer at 37°C overnight completely blocked the bands obtained using un-blocked antibody. Both blocked and un-blocked BORIS antibody was used at a dilution of 1∶100 in blocking buffer containing 2 ug/ml of peptide. (TIF) [file pone.0022399.s001.tif]

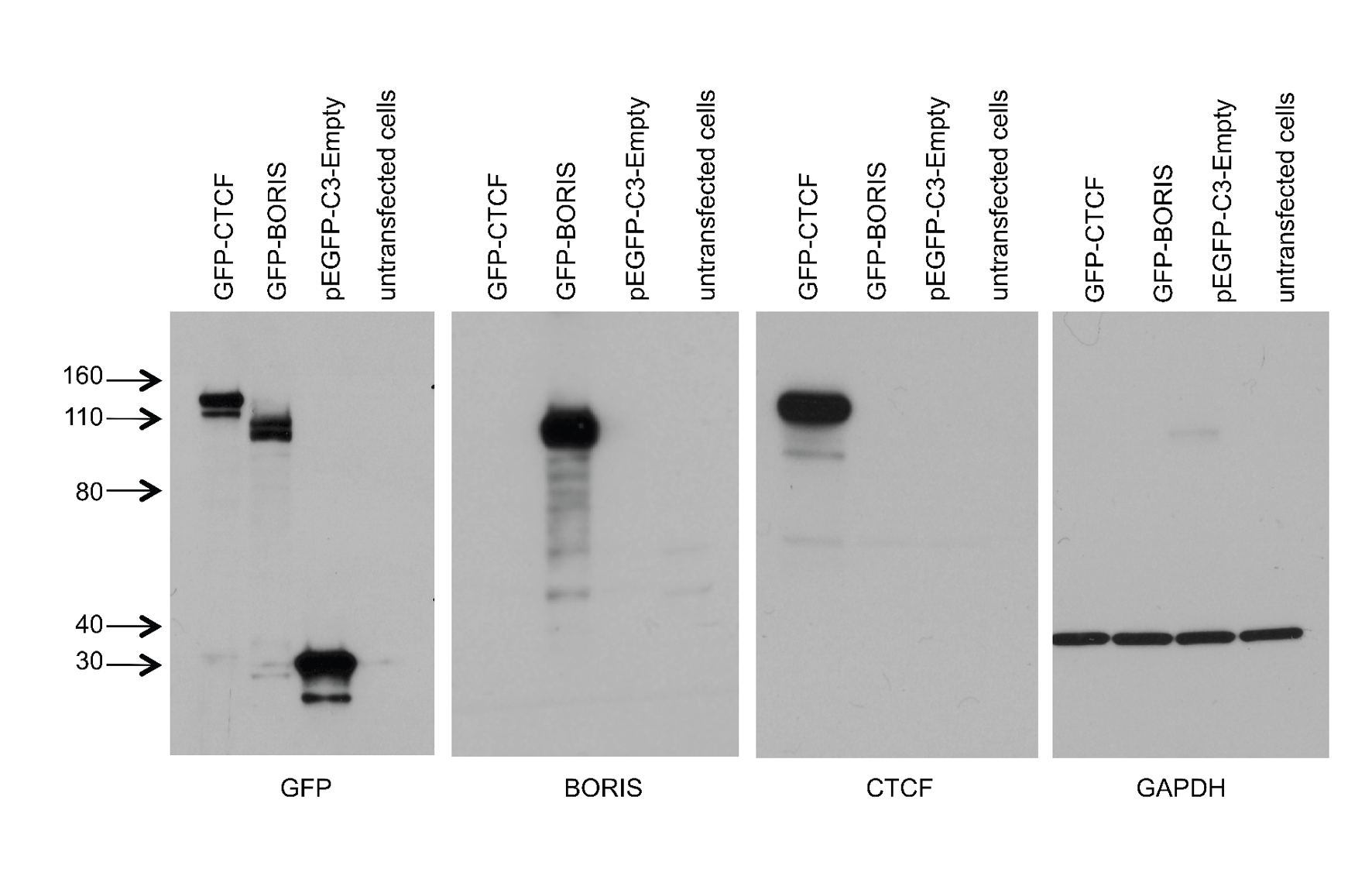

Supplement: Figure S2 — Western confirmation of BORIS antibody specificity. HEK293 cells transiently transfected with GFP-BORIS, GFP-CTCF or pEGFP-C3 empty vector (Clontech). Membranes probed with GFP, BORIS, CTCF or GAPDH antibodies. GFP-BORIS migrates at approximately 100 KDa, GFP-CTCF migrates at 140 KDa and GFP alone at 30 KDa. In comparison to the strong bands detected for GFP-BORIS, endogenous BORIS migrates at 50–70 KDa as much weaker bands. GAPDH was used as a control for loading differences. Invitrogen Novex®Sharp Pre-Stained Protein Standard, LC5800, was used for determination of band size. (TIF) [file pone.0022399.s002.tif]

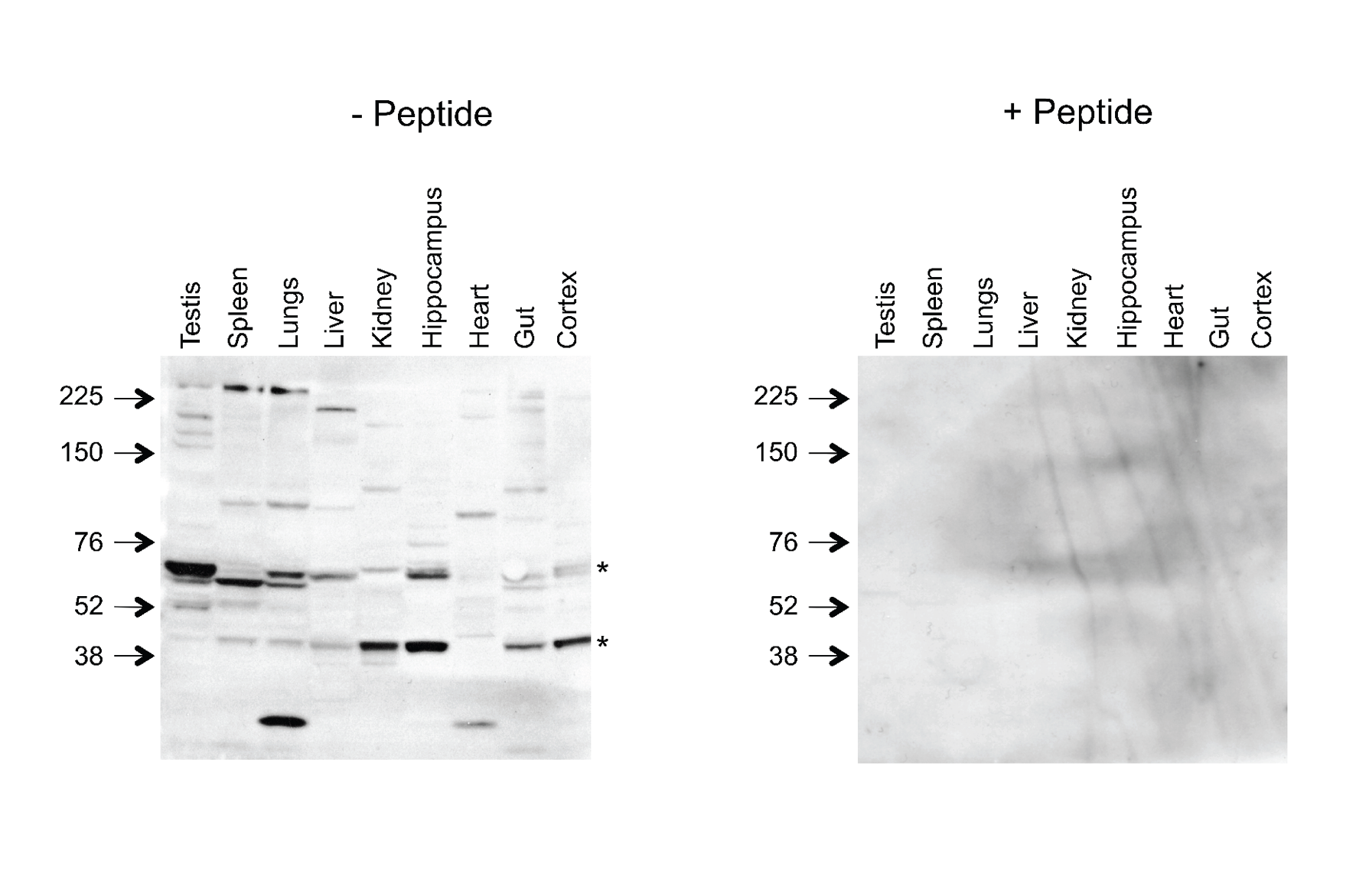

Supplement: Figure S3 — Antibody peptide competition in mouse tissues. Western blot analysis using BORIS antibody ab18337 (Abcam) with and without specific blocking peptide ab22203 (Abcam). Incubating 20 ug peptide with 10 ug BORIS antibody ab18337 (Abcam) in 200 ul TBST buffer at 37°C overnight completely blocked the bands obtained using un-blocked antibody. Both blocked and un-blocked BORIS antibody was used at a dilution of 1∶100 in blocking buffer containing 2 ug/ml of peptide. (TIF) [file pone.0022399.s003.tif]

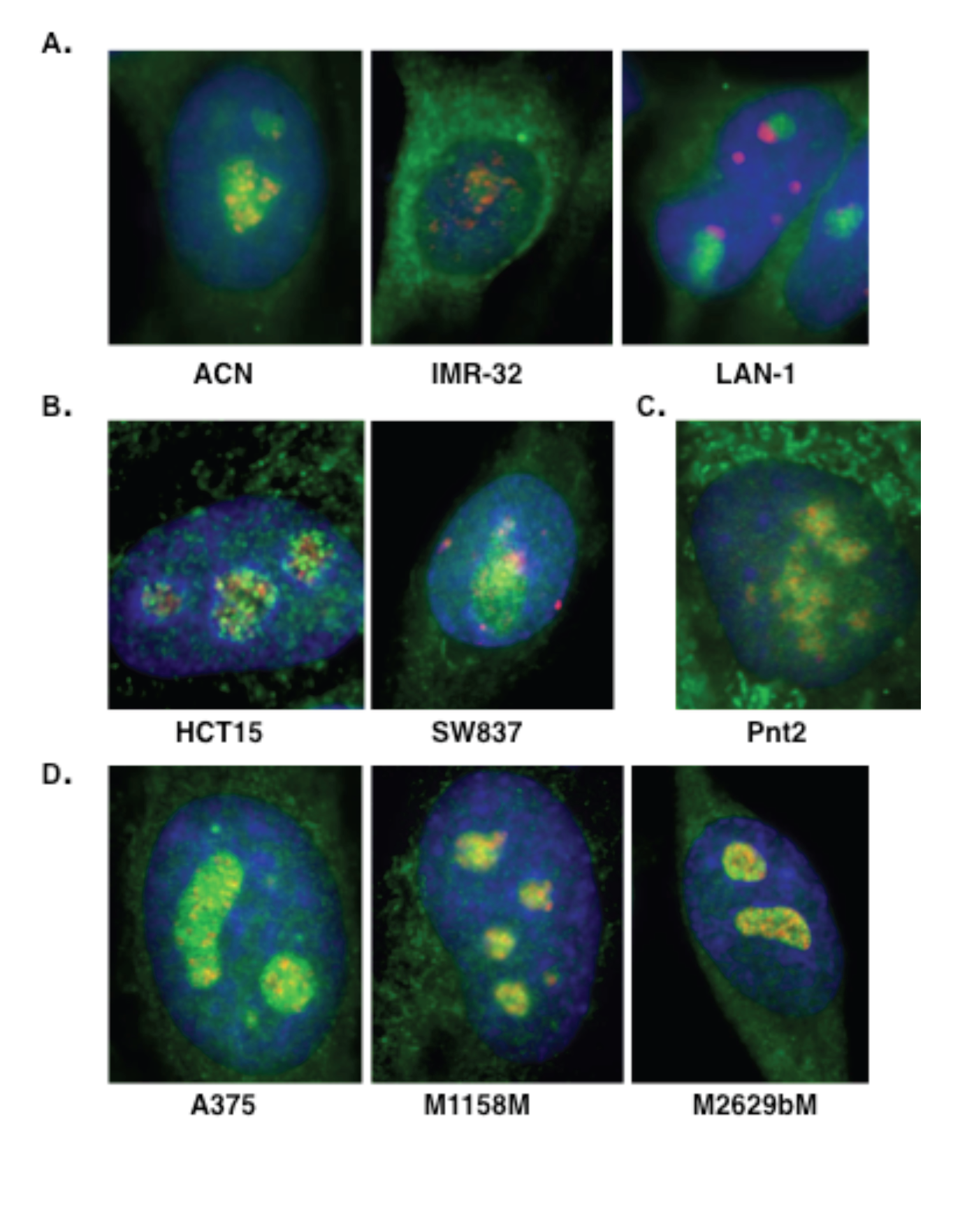

Supplement: Figure S4 — Distribution of BORIS in various cell lines. Immunofluorescence imaging of BORIS in green (Alexa 488) together with fibrillarin in red (Alexa 594), counterstained with 4′, 6-Diamidino-2-phenylindole (DAPI) in blue showing enrichment of BORIS within the nucleoli of A, neuroblastoma cell lines, B, colorectal cell lines, C, prostate cancer cell line and D, melanoma cell lines. Images are shown at 100x magnification. (TIF) [file pone.0022399.s004.tif]

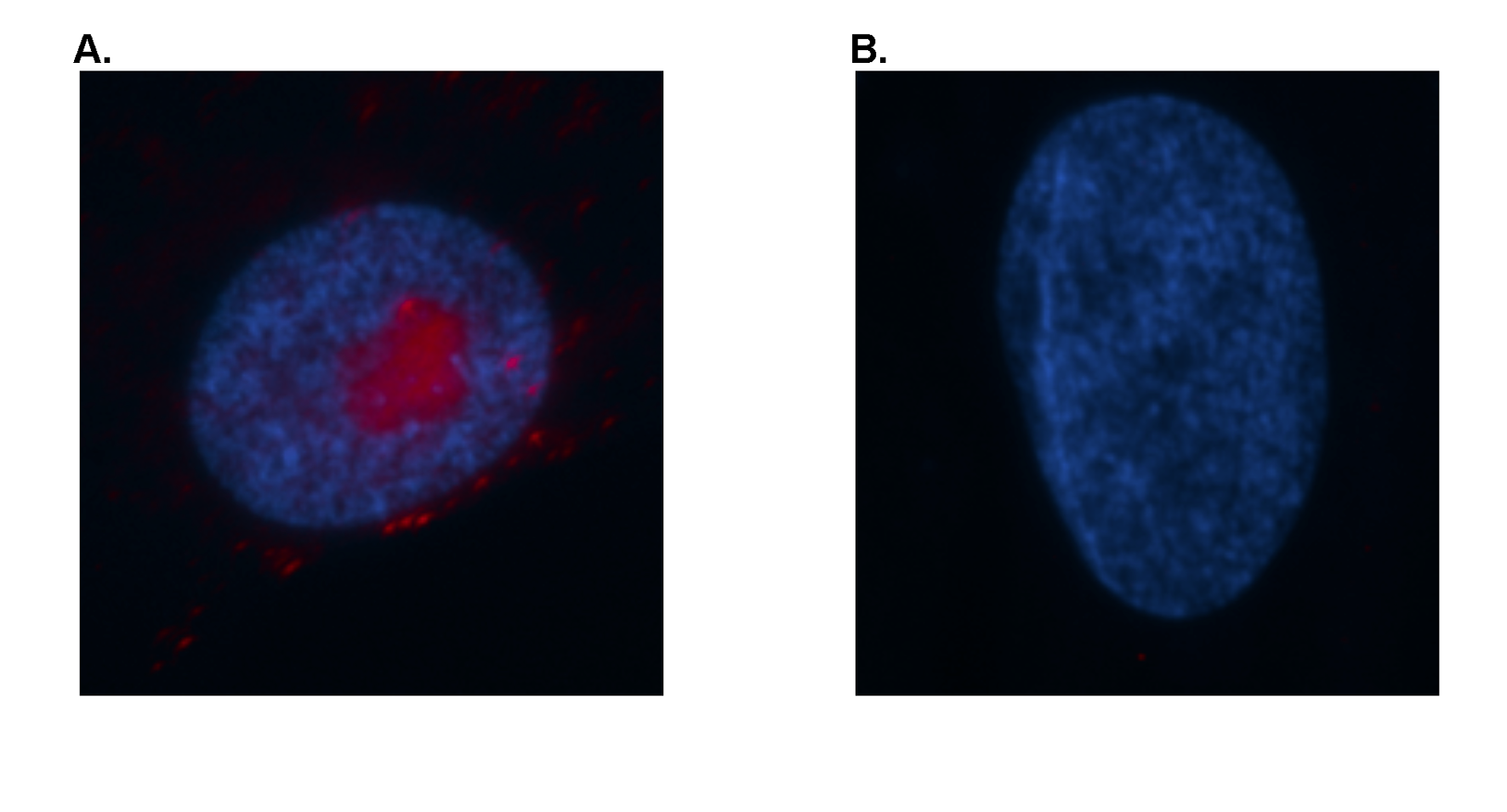

Supplement: Figure S5 — Antibody peptide competition in MRC5 cells. Immunofluorescence imaging of BORIS in red (Alexa 594) without peptide competition (A) and peptide neutralised BORIS antibody (B) counterstained with 4′, 6-Diamidino-2-phenylindole (DAPI) in blue. Images were obtained using identical exposure times and processing for a direct comparison of antibody versus antibody-peptide competition. Both blocked and un-blocked BORIS antibody was used at a dilution of 1∶10 in blocking buffer containing 2 ug/ml of peptide. Images are shown at 100x magnification. (TIF) [file pone.0022399.s005.tif]

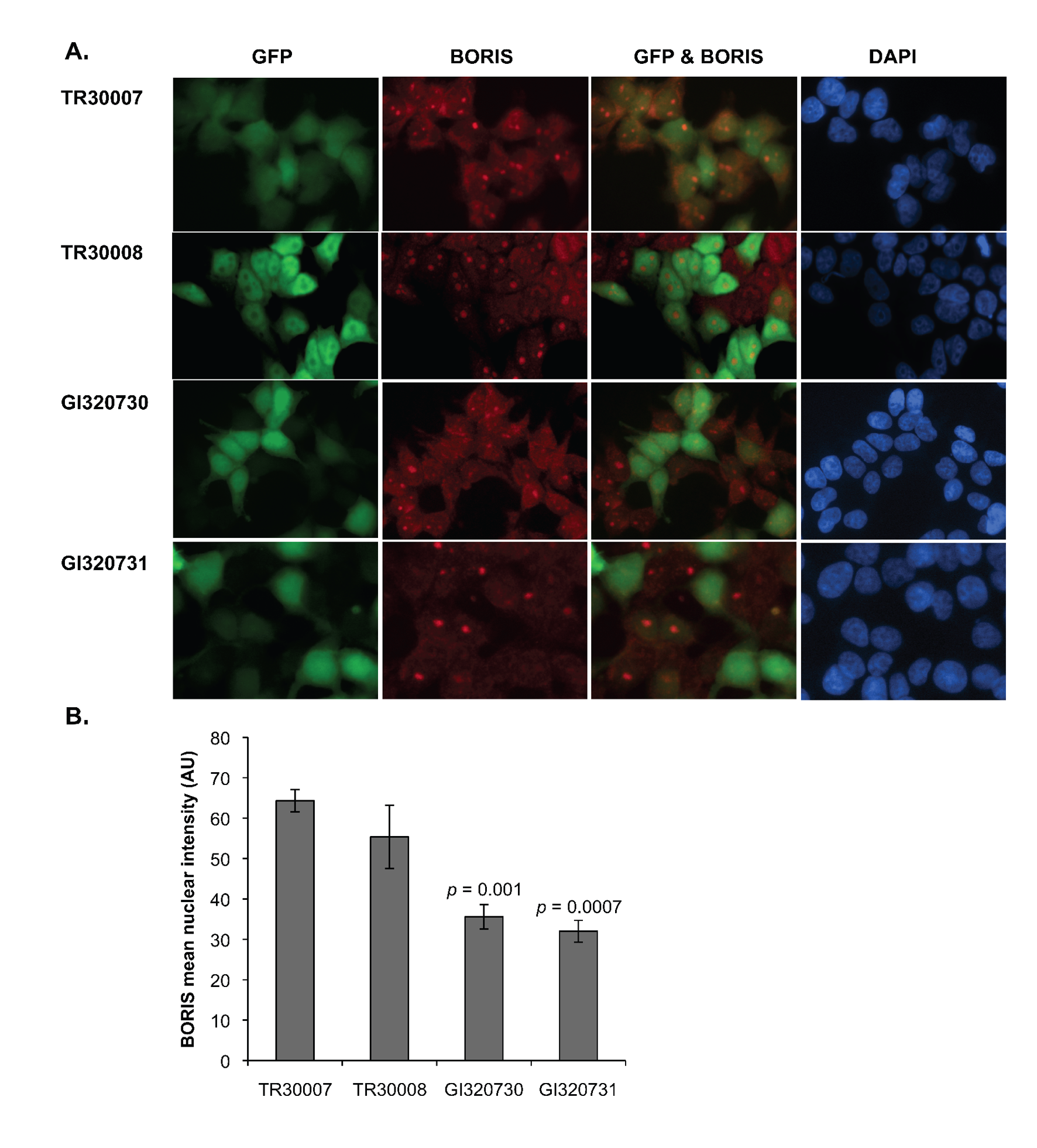

Supplement: Figure S6 — Immunofluorescence staining of HEK293T cells after BORIS knockdown. A, HEK293T cells transiently transfected with BORIS-specific shRNA, GI320730 (Exon 5 in NM_080618.2) or GI320731 (Exon 3, NM_080618.2), empty vector (TR30007) or scrambled control (TR30008). shRNA vectors express GFP (green) as a transfection reporter and cells were stained for BORIS (red) 72 hours after transfection. Cells were counterstained with 4′, 6-Diamidino-2-phenylindole (DAPI) shown in blue. Note the almost complete absence of nucleolar BORIS in GI320730 and GI320731 transfected cells (green). Images are shown at 40x magnification. B, Quantification of nuclear BORIS in A. Data is expressed as mean nuclear intensity (arbitrary units, AU) of all cells in 5 random fields with at least 30 cells per field and represents the data from 3 biological repeats. p-values in B were calculated by one-way ANOVA as compared to nuclear intensity in cells transfected with scrambled control. Error bars represent the standard deviation. (TIF) [file pone.0022399.s006.tif]

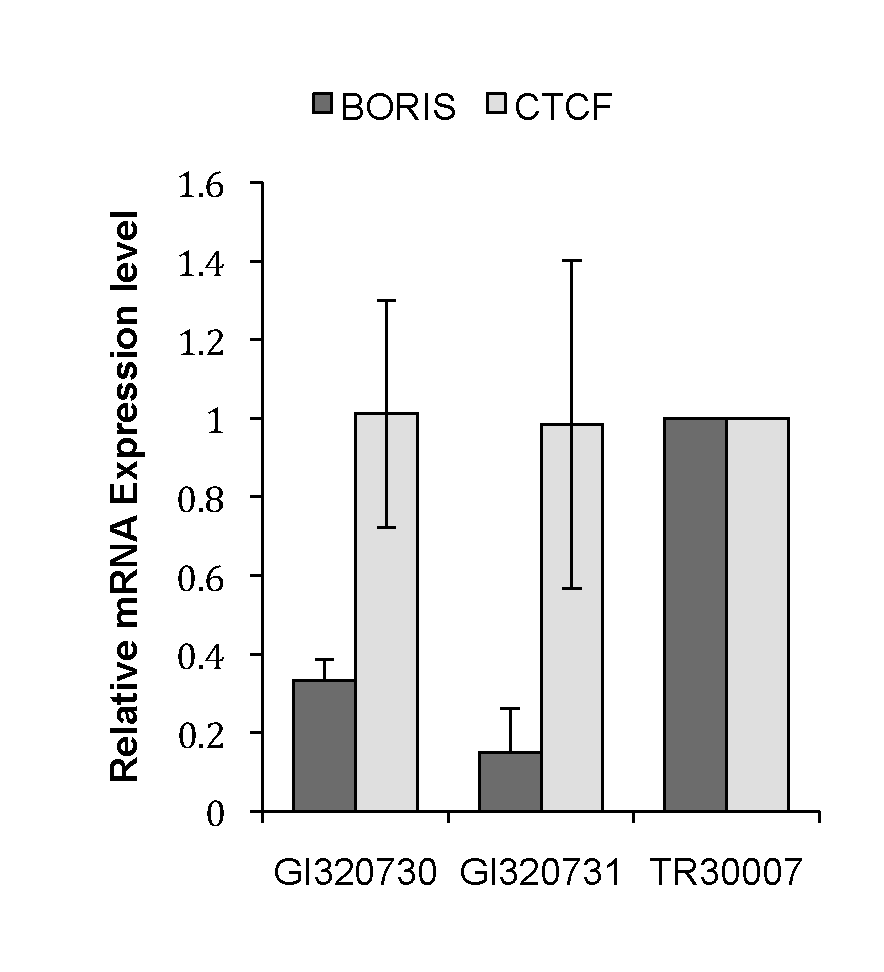

Supplement: Figure S7 — Transient knockdown of BORIS in HEK293T cells. Quantification of BORIS and CTCF transcripts in HEK293T cells after transient transfection with with BORIS-specific shRNA, GI320730 (Exon 5 in NM_080618.2) or GI320731 (Exon 3, NM_080618.2) or empty vector (TR30007). Data is normalised to GAPDH and empty vector set to 1. Error bars represent the standard deviation. (TIF) [file pone.0022399.s007.tif]
